# Dual and Opposite Roles of Reactive Oxygen Species (ROS) in Chagas Disease: Beneficial on the Pathogen and Harmful on the Host

**DOI:** 10.1155/2020/8867701

**Published:** 2020-12-10

**Authors:** Edio Maldonado, Diego A. Rojas, Sebastian Morales, Vicente Miralles, Aldo Solari

**Affiliations:** ^1^Programa Biología Celular y Molecular, Facultad de Medicina, Universidad de Chile, Santiago, Chile; ^2^Instituto de Ciencias Biomédicas, Facultad de Ciencias de la Salud, Universidad Autónoma de Chile, Santiago, Chile; ^3^Departamento de Bioquímica y Biología Molecular, Universidad de Valencia, Valencia, Spain

## Abstract

Chagas disease is a neglected tropical disease, which affects an estimate of 6-7 million people worldwide. Chagas disease is caused by *Trypanosoma cruzi*, which is a eukaryotic flagellate unicellular organism. At the primary infection sites, these parasites are phagocytized by macrophages, which produce reactive oxygen species (ROS) in response to the infection with *T. cruzi*. The ROS produce damage to the host tissues; however, macrophage-produced ROS is also used as a signal for *T. cruzi* proliferation. At the later stages of infection, mitochondrial ROS is produced by the infected cardiomyocytes that contribute to the oxidative damage, which persists at the chronic stage of the disease. The oxidative damage leads to a functional impairment of the heart. In this review article, we will discuss the mechanisms by which *T. cruzi* is able to deal with the oxidative stress and how this helps the parasite growth at the acute phase of infection and how the oxidative stress affects the cardiomyopathy at the chronic stage of the Chagas disease. We will describe the mechanisms used by the parasite to deal with ROS and reactive nitrogen species (RNS) through the trypanothione and the mechanisms used to repair the damaged DNA. Also, a description of the events produced by ROS at the acute and chronic stages of the disease is presented. Lastly, we discuss the benefits of ROS for *T. cruzi* growth and proliferation and the possible mechanisms involved in this phenomenon. Hypothesis is put forward to explain the molecular mechanisms by which ROS triggers parasite growth and proliferation and how ROS is able to produce a long persisting damage on cardiomyocytes even in the absence of the parasite.

## 1. Introduction

Protozoan parasites from the *Trypanosomatidae* family are unicellular and diploid organisms, which cause neglected diseases in many regions of the world, mainly in developing countries with tropical and subtropical climates. Chagas disease is a neglected tropical disease, which affects over seven million people around the world. It is caused by the unicellular flagellate protozoan *Trypanosoma cruzi* and affects over 8 million people worldwide, causing approximately 50,000 deaths each year [1, 2]. Another 70-100 million people living in endemic areas are at risk of infection [1, 2]. For most of the Latin American countries, it is one of the main public health problems. Many other factors such as migration events are increasing the number of diagnosed cases in nonendemic regions such as Europe, North America, and Western Pacific areas [3].

Chagas disease is transmitted by blood-sucking bugs of the subfamily *Triatominae*. The disease has two successive phases. The first phase is an acute one characterized by high parasitemia and a second chronic phase is usually with chronic cardiomyopathy. Most of the infected individuals in the second phase (60-70%) never develop chronic symptoms or signs associated with the disease; however, the rest of the patients (30-40%) will show signs of heart impairment. Chagas disease displays a variety of clinical forms, and the possible outcomes of this disease involve interplay between environmental and genetic factors associated with both the host and the infecting parasite [4, 5]. During the initial acute phase, the infecting parasites (blood trypomastigotes) can invade tissues and they mainly multiply inside the macrophages leading to high parasitemia and inflammation. Most individuals then circumvent this acute phase of infection and enter in a new phase, which is characterized by a low parasite numbers in the blood and no apparent pathological features. However, for 30-40% of patients, a chronic phase appears 20-30 years later and is characterized by low parasitemia but an increased tissue injury, which results in severe digestive and/or cardiac damage that can be lethal if patients are not treated [6]. Two drugs have been commonly used to treat *T. cruzi* infections for the last 40 years, which are benznidazole and nifurtimox [6, 7]. They are believed to exert their trypanocidal activity through the bioreduction of the nitro group by trypanosomal type I reductase and followed by covalent linkage of the reduced drug to internal thiols which can form toxic adducts on the parasite DNA.

The *Trypanosomatidae* family belongs to the *Kinetoplastida* order, which contains a single and unusual mitochondrial DNA or kinetoplast DNA containing several dozens of maxicircle copies, which code for proper mitochondrial functions and several thousand of minicircles copies coding for guide RNAs, which edit mitochondrial mRNA; all of them are concatenated in a single net that occupies the only one mitochondrion in this flagellate [8, 9].

*T. cruzi* goes through extensive morphological and biochemical changes during its life cycle, which alternates between the vertebrate and the invertebrate hosts (*Triatomine* bugs). Noninfective epimastigotes proliferate in the hindgut of the insect vector, which are haematophagous bugs. Once in the rectum of the insects, parasites differentiate into the nondividing infectious metacyclic trypomastigotes [10, 11]. These parasites are excreted with the insect excrements after they feed on the mammalian host and can infect this host by passing through different mucous membranes, such as the stomach or skin lesions produced during or after the insect blood meal. These infective metacyclic forms invade host cells, mainly macrophages, where they transform into the replicating intracellular amastigote stage [10, 11]. After multiplying by binary division in the cytoplasm, amastigotes differentiate into infective nonreplicating trypomastigotes. Both forms are released into the bloodstream of the mammalian host upon host cell lysis. Subsequently, trypomastigotes can penetrate other nucleated cell types, including skeletal and cardiac muscle cells (cardiomyocytes), which depend on the immune competent state of the host, or they can be taken up again by the insect during a blood meal, starting a new infecting cycle [10, 11].

In 1968, the reactive oxygen species (ROS) produced by phagocytes during respiratory burst was proposed as the killing mechanism of pathogens. By the late 1970 and early 1980, it was proposed and assumed that ROS from the macrophages was the responsible of the killing of *T. cruzi*; however, a few years ago (2012), the old paradigm has shifted. *T. cruzi* can live inside the macrophages under oxidative stress conditions; moreover, antioxidants are detrimental for its growth. In this review, we will summarize the mechanisms by which *T. cruzi* can deal with the host-oxidative assault, and also we will discuss the most important work demonstrating that ROS produced by the host cells in response to the infection can promote *T. cruzi* cell growth and differentiation and how ROS produced by the infected cell contributes to the progressive oxidative damage produced in Chagas heart disease at the chronic stage.

### 1.1. *Trypanosoma cruzi* and Oxidative Damage

*T. cruzi* is a well-equipped organism to deal with endogenous and/or exogenously produced oxidants. As an obligatory intracellular parasite, *T. cruzi* must be able to withstand its own endogenous toxic metabolites, produced during its aerobic mitochondrial metabolism, and also must cope with the oxidative burst from the host immune system, which includes several reactive nitrogen and oxygen species (RNS and ROS, respectively). Moreover, they can use ROS as a signal for cellular growth and proliferation. In this way, *T. cruzi* has evolved mechanisms to deal and use oxidative signals in their own benefit. This fact is reflected by the efficient and well-compartmentalized antioxidant network that the parasite uses in the detoxification of ROS and RNS species produced during parasite-host cell interactions and the efficient use of ROS to grow and proliferate.

In *T. cruzi*, the antioxidant defenses are composed for several enzymes and nonenzymatic redox active molecules, which are located mainly in the mitochondria, endoplasmic reticulum, glycosomes, and the cytosol [12–14]. The final electron donor for the enzymatic reactions is NADPH derived from the pentose pathway. In such a way, the reducing equivalents from NADPH are delivered to a variety of enzymatic detoxification systems of the parasite, mainly through the dithiol trypanothione [T(SH)_2_, N1, N8-bisglutathionylspermidine] and the thioredoxin homologue named tryparedoxin (TcTXN) which are low-molecular-weight dithiol proteins belonging to the thioredoxin oxide reductase family or glutathione (GSH). Thus, despite the lack in the parasite of an enzyme-mediated pathway to detoxify H_2_O_2_, they rely mainly on trypanothione (TR) [12–14]. Trypanothione is synthetized by the trypanothione synthetase (TcTS) in an ATP-dependent reaction in which two molecules of GSH are covalently linked to spermidine. The reduced state of trypanothione is maintained by the NADPH-dependent flavoenzyme trypanothione reductase (TcTR) [15]. Trypanothione also provides reducing equivalents to the ribonucleotide reductase to generate deoxyribonucleotides for DNA synthesis [12–14]. In addition, *T. cruzi* has the complete biosynthetic pathway for ascorbate that can act both as a nonenzymatic antioxidant and as the reducing substrate for ascorbate-dependent hemoperoxidase (TcAPX) [12]. Other low-molecular-weight thiols such ovothiol A, which is found in the replicative stages but not in the blood forms of *T. cruzi*, might also possess antioxidant roles.

*T. cruzi* possesses five different peroxidases, which can differ in their specificity and subcellular location [16]. Two of these, the mitochondrial and cytosolic *T. cruzi* tryparedoxin peroxidases TcMPX and TcCPX, respectively, belong to the peroxiredoxin family of proteins, which are able to detoxify H_2_O_2_ short-chain organic hydroperoxides and peroxynitrite, which are as efficient as catalase and selenium-dependent glutathione peroxidase in H_2_O_2_ detoxification [16]. Another peroxidase, the ascorbate-dependent heme-peroxidase (TcAPX), is located in the endoplasmic reticulum and is able to confer resistance against H_2_O_2_ using ascorbate as reducing substrate. Other two peroxidases, which show similarity to glutathione-dependent peroxidases, are glutathione-dependent peroxidase I (GPX-I), which is located in the cytosol and glycosome, and the glutathione-dependent peroxidase II (GPX-II), which is present in the endoplasmic reticulum; however, they are unable to act against H_2_O_2_. Interestingly, a catalase enzyme is absent in *T. cruzi*; however, this enzyme is present in other kinetoplastides, which parasitize insects, and is also present in almost all aerobic organisms [17]. Efforts to answer this puzzling observation have been done with the generation of a *T. cruzi* cell line expressing a heterologous catalase (from *E. coli*), since it might be possible that O_2_^·-^ and/or H_2_O_2_ can act as intermediates in cellular signaling pathways. The artificial expression of catalase increases the resistance to H_2_O_2_ compared to wild-type cells but reduces the parasite ability to adapt under a low H_2_O_2_ dose environment, suggesting that catalase has an effect in the parasite adaptation ability, which is promoted by pretreatment of the parasites with low doses of the oxidant [16]. Moreover, the expression of the heterologous catalase is able to reduce the levels of trypanothione reductase, but increases the levels of superoxide dismutase, which results in higher levels of residual H_2_O_2_ after the treatment with this oxidant [17].

In the antioxidant network dependent on trypanothione, this molecule is reduced by the enzyme TcTR which transfers the reducing equivalents from NADPH via trypanothione to tryparedoxin TcXNI, which is the source of reducing equivalents for cytosolic tryparedoxin peroxidase TcCPX [12–14]. The rate-limiting step in the antioxidant trypanothione-dependent system is the interaction between trypanothione and TcXN. The TcTXN possesses an important role to protect the parasites against fluctuating levels of ROS which is generated during the physiological process, since it can be modulated by the exposure of the parasites to H_2_O_2_ and has a higher expression in culture-derived trypomastigotes treated with H_2_O_2_, and its expression levels are higher in strains more resistant to the oxidative damage caused by this compound [18–21]. This enzyme is also released into the culture medium; however, the mitochondrial TcMPX is not, but the reason for this observation is not known. TcMPX is located inside the mitochondrion, toward the cell periphery in close contact with the kinetoplast DNA, and therefore might protect mitochondrial DNA from peroxide-mediated damage [20, 21]. It has been hypothesized that TcMPX acts in a similar way, as TcCPX, since both can act on the same substrates; however, trypanothione TcTR and TcXN have not been found inside the *T*. *cruzi* mitochondrion. So far, the mechanism by the mitochondrial NADPH which is regenerated inside the mitochondrion remains unclear. It seems to be that *T. cruzi* relies only on TcCPX to detoxify the H_2_O_2_ generated inside the mitochondrion [14, 18, 19].

**Figure 1:**
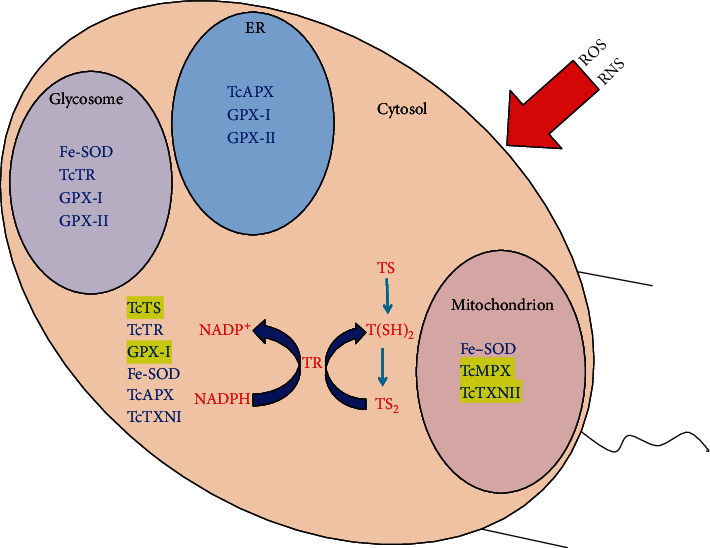
Antioxidant defenses location in *T. cruzi*. The dithiol trypanothione [T(SH)_2_] and the *T. cruzi* antioxidant defense enzymes are distributed into the ER, glycosome, mitochondrion, and cytosol. ROS: reactive oxygen species; RNS: reactive nitrogen species; TR: trypanothione; TcTS: trypanothione synthetase; TcTXN: tryparedoxin; TcTR: trypanothione reductase; TcAPX: ascorbate-dependent hemoperoxidase; TcMPX/TcCPX: tryparedoxin peroxidase; GPX-I: glutathione-dependent peroxidase I; GPX-II: glutathione-dependent peroxidase II; Fe-SOD: iron-dependent superoxide dismutase.

Two thiol-dependent tryparedoxin peroxidases (TcTXNI and TcTXNII) exist in *T. cruzi*. Tryparedoxins are dithiol proteins of low molecular weight, which belong to the thioredoxin family of proteins. TcTXNI can interact with several cytosolic endogenous proteins involved in the antioxidant system, energy metabolism, and protein translation machinery [14, 18–20]. One of the interacting partners, as expected, is TcCPx. On the other hand, TcTXNII is a mitochondrial transmembrane protein and is anchored to the surface of this organelle and also on the surface of the endoplasmic reticulum with the redox active center exposed towards the cytosol. TcTXNII is able to interact with protein members of the antioxidant system, cytoskeleton, energy metabolism, and protein synthesis machinery. Also, both TcCPx and TcMPX were found to be able to interact with TcTXNII [18, 19]. However, it is believed that tryparedoxins are not required for mitochondrion redox metabolism and TcMPX might use a reduction system that does not require tryparedoxins. However, it has been found that TcTXNI and TcXNII peroxidases can efficiently reduce H_2_O_2_ and peroxynitrite *in vitro* [22, 23] and this could contribute to their role as virulence factors reported *in vivo*. It is also likely that thiol-dependent tryparedoxin peroxidases could play a minor nonessential role in the parasite antioxidant defense system, and perhaps they play an important role in other biological processes. An overview of the enzymes involved in the oxidative stress and their cellular location is displayed in Figure 1.

The mitochondrion is the principal site for oxidant formation during the aerobic metabolism. Those oxidants can be able to exert damage and/or participate in signal transduction processes. Superoxide radical, generated by electron leakage from the respiratory chain (mainly at the complex III), is metabolized by an iron-dependent superoxide dismutase (FeSOD-A) [24] to produce H_2_O_2_, an oxidant that must be further catabolized. Meanwhile, the nitric oxide (NO) which reaches the mitochondria can inhibit parasite mitochondrial respiration (at the complex IV) enhancing the generation of O_2_^·-^ (by complex III) which produces intramitochondrial peroxynitrite formation (ONOO^−^). This process can outcompete the dismutation of O_2_^·-^ by the enzyme FeSOD-A [24]. To overcome this, there exists a typical two-cysteine peroxiredoxin located at the mitochondrial matrix (TcMPX) which efficiently reduces and detoxifies the ONOO^−^ and H_2_O_2_ to NO_2_ and H_2_O, respectively [25]. On the other hand, in the endoplasmic reticulum, two distinct peroxidases, which are named ascorbate-dependent heme-peroxidase (TcAPX) [26] and glutathione-dependent peroxidase II (GPX-II), can metabolize H_2_O_2_ and lipid hydroperoxides, respectively [13, 14]. Meanwhile, the glycosomes, which are specific trypanosomal organelles and the places where the first set reactions of the glycolysis pathway occur, contain Fe-SOD, glutathione-dependent peroxidase I (GPX-I), and GPX-II [13, 14]. The enzyme GPX-I can metabolize H_2_O_2_; however, the enzyme GPX-II has a more restricted specificity directed towards lipid hydroperoxides. Finally, the cytosolic antioxidant defenses are supplied by the presence of GPX-I, Fe-SOD, and also by the cytosolic peroxiredoxin TcCPX. The relevance of each of those components of the parasite antioxidant defenses has been demonstrated by the increased resistance against H_2_O_2_/ONOO^−^ treatment, which is afforded when those enzymes are overexpressed in *T. cruzi* epimastigote cells.

In spite of the antioxidant defenses of the parasites that might look undefeatable, the macrophage-produced peroxynitrite can damage and control *T. cruzi* proliferation [27, 28]. Cytotoxic actions of peroxynitrite against parasites include alteration of Ca^2+^ homeostasis which compromises cellular energetic charge and mitochondrial physiology and can mediate either necrotic or apoptotic death pathways, depending on the severity of the oxidative insult [27, 28]. The ultimate success of the parasite to infect the host will depend on the macrophages' ability to destroy the infecting parasite, the host immune system, and the virulence factors of the parasite.

### 1.2. Oxidative Stress in the Acute Phase of Chagas Disease

In order to get a successful infection, the metacyclic trypomastigotes are required to invade the host cells. In the vertebrate host, resident macrophages, present at the parasite invading site, are among the first professional phagocyte cells to be invaded *by T. cruzi*; therefore, they are key players in the infection control [28–31]. The naive macrophages (noncytokine primed) internalize infecting metacyclic trypomastigotes into the phagosome vacuole and this event activates the membrane-associated NADPHox [31]. The phagocytic stimuli event makes that cytosolic subunits of the NADPHox can associate with the other plasma membrane counterparts and can form an active complex, a flavoenzyme which is responsible for the generation of large amounts of O_2_^·-^ directed towards the internalized invading parasite [31]. Since the O_2_^·-^ has an anionic nature that does not diffuse through membranes, its action spectrum is reduced only at the production site. The O_2_ radical can spontaneously produce H_2_O_2_ or can suffer an enzyme-mediated dismutation to H_2_O_2_ by the action of superoxide dismutase, which produces an oxidant with higher diffusional capacity. Some metal transition ions in the presence of H_2_O_2_ are able to generate hydroxyl radical (OH^·^), an oxidant with high reactivity, but displays poor selectivity against cellular targets. Although resident naïve macrophages can produce ROS at the infection site, this by itself is not sufficient to clear the parasite inside the macrophage phagosome vacuole. Macrophages produce proinflammatory cytokines such as IL-12, IFN*γ*, and TNF-*α*, and this results in the priming of macrophages. This event can lead to the induction of the inducible nitric oxide synthase (iNOS) system, which is able to generate high amounts of NO that can be maintained during 24 h, releasing from the macrophages all the whole oxidant cytotoxic power [29, 30]. The NO produced in the cytosol is a hydrophobic radical that diffuses to the phagosome vacuole where it reacts with O_2_ to produce peroxynitrite (ONOO^−^), which is a powerful oxidant and cytotoxic effector molecule, which can kill a variety of pathogens including trypanosomes [32, 33]. However, ONOO^−^ is a short-lived molecule but can cause damage, by direct reactions via one or two electron oxidation mechanisms to several molecules such as thiols and metal centers. Therefore, the ONOO^−^ production by the macrophages during the first hours of infection is fundamental to control the intraphagosomal parasite survival, before the replicative amastigotes can reach the safe cytoplasmic environment [33].

During the first week of infection, several proinflammatory cytokines are produced, preferentially IFN*γ* by the host natural killer cells. This event is important, since the cytokine production can prime naive macrophages to induce iNOS, which will generate trypanocidal oxidative mediators [16]. Weeks after, the inflammatory cytokines are produced by the host T lymphocytes and in the mean, while the adaptive immune response against the parasite is mounting. The IL-12/IFN*γ*/iNOS axis has a pivotal role in the control of *T. cruzi* infection and also has important implications for the outcome in Chagas disease [34–36]. These observations have been well documented in conclusive experiments where it was used knockout mice for IFN*γ*, IL-12, and iNOS [35, 36].

In a very recent study to investigate the role of NOX2-derived ROS, it was found that in *T. cruzi*-infected mice, which lack a functional NOX2 oxidase, the cardiomyocytes displayed a proarrhythmic phenotype in the acute phase of experimental Chagas disease [37]. Moreover, it was found that an imbalance between the production of ROS and NO can increase the cellular electromechanical dysfunction which can lead to severe arrhythmias. However, it is possible to inhibit NO production, by a treatment with a nonspecific NOS inhibitor, and attenuate the imbalance between ROS and NO, which leads to a recovery of the cardiomyocyte electrical function and to a decrease of in vivo arrhythmia [37]. The effect of lack of NOX2 oxidase on the heart cells at the chronic phase of Chagas disease has not been described yet.

### 1.3. ROS as a Promoter of *T. cruzi* Infection

Upon infection by *T. cruzi*, host cells can respond in producing several factors, such as NOS, ROS, and cytokines, among others. As we have discussed, NO and ROS are combined to produce ONOO, which is a powerful killing agent against phagocyted parasites. For several decades, it was believed that ROS was used to eliminate the invading parasites. However, recent evidences have suggested that the parasite proliferation is stimulated in oxidative environments [38–41]. Whether or not an oxidative environment provides the ideal conditions for *T. cruzi* growth is still a matter of discussion. However, Goes et al. [39] have proposed that ROS might play a dual role in the *T. cruzi* life cycle, in which high levels of ROS (oxidative environment) are necessary for proliferation, while a low level of ROS (reduced environment) is obligatory to promote metacyclogenesis.

The recent evidence suggests that ROS are able to produce signals to stimulate *T. cruzi* growth [39, 40]. However, it must be noted that counterevidence for the role of ROS as promoter of cell growth also exists. It has been demonstrated that oxidative stress generated by *T. cruzi* infection can lead to an increase in the replication rate of the parasite [38–40]. Also, it has been proposed that the reason for this finding is the availability of iron, from heme, to be used by the parasite, since *T. cruzi* is auxotrophic for heme, which is an important cofactor for several enzymes involved in key biological processes and also a source of iron [38]. Heme-induced ROS is able to stimulate epimastigote cell proliferation [38] and recently has been shown that heme regulates the activity of *T. cruzi* TcK2 kinase [42]. TcK2 is an eIF2A kinase, which phosphorylates the alpha subunit of initiation factor 2 involved in protein synthesis. The phosphorylation of this factor by TcK2 decreases translation and in the absence of heme TcK2 is active and promotes cell growth arrest, which leads to the differentiation of proliferative amastigote cells into infective nonproliferative trypomastigote cells [42].

It has been largely documented that an increased ROS production can produce oxidative damage to the host during both the acute and chronic phases of Chagas disease [40, 43, 44]. The increased ROS production has been ascribed to NADPHox2 activation in the unstimulated infected host macrophages [40, 44]. Thus, it might be possible that reduction of the oxidative stress could reduce tissue damage in Chagas disease. Indeed, NADPHox2 enzyme inhibition can ameliorate *T. cruzi*-induced myocarditis during Chagas disease [40]. Even though it is expected that antioxidants can increase parasite burden, however, they are unable to do so. Moreover, the treatment of mice, at the beginning of the infection, with antioxidant cobalt protoporphyrin (CoPP), reduces parasite burden. CoPP activates the nuclear factor erythroid-derived 2-like 2 (NRF2), which in turn increases the expression of heme oxygenase (HO-1) [39, 45]. NRF2 is a transcriptional activator that controls gene expression of antioxidant enzymes, when cells are under oxidative stress conditions [39]. The antioxidant was able to reduce parasitemia, tissue parasitism, and reduced macrophage parasitism. Prooxidants were able to promote macrophage parasite burden, indicating that the redox state of the cell is important for *T. cruzi* infection [45, 46]. The effects of CoPP were not directly on the parasites, instead were dependent on the induction of the NRF2/HO-1 pathway. Moreover, either the overexpression of NRF2 or HO-1 can reduce macrophage parasite burden [40]. One of the first studies is launched to systematically assess the effects of oxidative stress on the parasite burden during acute *T. cruzi* infection, contradicted the paradigm of ROS as trypanocidal agent [40, 45]. In this study, nrf2 and ho-1-knockout mice were produced to assess the parasite burden in macrophages derived from those knockout mice. The treatment with CoPP reduced the parasite burden in infected macrophages from ho-1-knockout mice; however, those derived from nrf2-knockout mice did not [40]. However, transfected THP-1 human macrophages with either ho-1 or nrf2 genes, before infection, have reduced parasite burden [40]. Those observations indicate that the effects of CoPP are dependent on the redundant action of the NRF2-controlled genes, since this transcription factor controls the expression of several genes involved in the antioxidant defense. Incubation of infected macrophages with several antioxidants or with the enzyme polyethylene glycol-conjugated superoxide dismutase (PEG-SOD) reduces parasite burden while respiratory burst-inducer phorbol 12-myristate 13-acetate (PMA), H_2_O_2_, and prooxidant paraquat can increase the parasite burden [44]. It has been observed that the treatment of *T. cruzi* cells with low concentrations of H_2_O_2_ can improve the proliferation and they increase the resistance against sublethal doses of H_2_O_2_ which can cause oxidative stress [46]. In those studies, it was also observed that H_2_O_2_ induce the increasing levels of TcCPX and this may be an initial cell attempt to promote detoxification [47]. These observations suggest that adaptation to oxidative stress is assured when cells are first exposed to low concentrations of H_2_O_2_ and are afterwards exposed to higher concentrations at H_2_O_2_. Another observation is that *T. cruzi*-infected macrophages, which lack NADPHox, have reduced parasite burden than their wild-type counterparts; however, when those infected macrophages are incubated with H_2_O_2_, the infection index increases [40]. That indicates that H_2_O_2_ supplies an additional proliferation stimulus to *T. cruzi*, which is lacking in macrophages that do not have the NADPHox enzyme. From those studies, we can have a general picture that emerges and shows that ROS is able to fuel *T. cruzi* infection in macrophages during the early acute phase. Whether or not ROS could reach high concentrations inside the invaded macrophages, capable of killing or restraining amastigote growth instead of stimulating their proliferation, is still an unknown matter and it must be studied further on. When infected macrophages are incubated with sublethal doses of up to 100 *μ*M H_2_O_2_, this promotes a more intensive amastigote proliferation [47]; however, it is unknown the actual concentration of H_2_O_2_ that can be reached inside the cytosol after this treatment. It is known that the cytosolic concentration of H_2_O_2_ during macrophage respiratory burst is within the 1-4 *μ*M range [47].

The role played by ROS-producing macrophages at the chronic stage of infection has not been evaluated yet, but it seems likely that at this stage of the disease, most of them are activated by IFN*γ* to induce the iNOS pathway to produce NO and ONOO with trypanocidal activity. On the other hand, the idea that ROS promotes *T. cruzi* parasitism might help to explain several results reported earlier in the literature. Cruzipain is the major cysteine protease from *T. cruzi*, and the expression of this an enzyme increases the susceptibility of macrophages to *T. cruzi* infection [48] and it is the major inducer of NADPHox2 activation, which can produce ROS during macrophage infection and in such a way induces parasite cell growth. Cruzipain is able to cleave chemokines and this activity might be in part responsible for the increased susceptibility of macrophages to infection, due to the lack of macrophage activation. On the other hand, the lack of the gene of signaling lymphocytic activation molecule family member (Slamf1) is a condition that reduces NADPHox2 activation in myeloid cells and was found that it increases resistance to *T. cruzi* infection [49]. Aryl hydrocarbon receptor (AhR) is a ligand-activated transcription factor that plays important roles in the immune response controlling the expression of genes involved in immune responses, including the activation and differentiation of specific T cell subsets and antigen-presenting cells, and also regulates ROS production in response to infections. AhR is a suppressor of cytokine signaling pathway in the spleen and heart and as consequence influences NO and ROS production. Indeed, infected AhR-knockout mice displayed significantly reduced parasitemia, inflammation, and fibrosis of the myocardium [50]. This was associated with an anticipated increased immune response characterized by increased levels of inflammatory cytokines and low ROS production. *In vitro*, AhR deficiency caused impairment in parasite replication and decreased levels of ROS production [50]. Also, the treatment of infected mice with curcumin [51] or melatonin [52], which both are able to activate the host antioxidant defenses, reduced parasite burden in blood and heart tissue. Taken altogether, those results are in complete agreement with the general idea that ROS can promote *T. cruzi* infection.

However, the molecular mechanisms by which ROS can promote *T. cruzi* infection have to be still fully understood. It has been shown that antioxidants upregulated the expression of H-ferritin (H-Ft), a cytosolic protein that can bind iron and ferroportin-l (Fpn-1), a channel that allows iron efflux [53]. The increased expression of those proteins decreases the labile iron pool (LIP) in macrophages [53, 54]. The decreasing of LIP also decreases the parasite burden, while the increase of the LIP has the opposite effect. Iron also increased the parasite burden of infected *gp9l* macrophages (which lack NADPHox) to an extent similar to that found in wild-type macrophages. These results can be interpreted that regulation of LIP is the main mechanism underlying the increase in macrophage parasitism produced by oxidative stress [55].

As mentioned before, several research groups have explored the idea that the cellular oxidative environment by itself is a direct growth stimulus for *T. cruzi*. It is most likely that *T. cruzi* profits from the stress generated from ROS, and this might provide an adaptive evolutionary advantage for the parasite. Many evidences point towards that direction: parasites can proliferate in response to H_2_O_2_ [47] and the increased growth of epimastigotes in response to H_2_O_2_ involves a calmodulin-dependent protein kinase II CAMKII-like enzyme in the proliferation pathway and a cascade of events including AMP kinase in the host [38, 56, 57] and also the mitochondrial ROS produced into the mitochondria, which results from heme metabolism and enhances the parasite growth [49]. As mentioned earlier, heme also controls the activity of TcK2 and in turn the process of translation in the parasite. Parasites lacking TcK2 lose this differentiation capacity, and heme is not stored in reserve organelles, remaining in the cytosol. On the other side, TcK2 null cells display growth deficiencies, accumulating H_2_O_2_ driving the generation of ROS [42]. The augmented level of H_2_O_2_ occurs as a consequence of increased superoxide dismutase activity and decreased peroxide activity. These observed phenotypes can be reverted by the reexpression of the wild-type TcK2 but not for a dead mutant version of the kinase [42]. These findings indicate that heme is a key factor for the growth control and differentiation through regulation of an unusual type of eIF2*α* kinase in *T. cruzi* [42, 58].

The effects of antioxidants, oxidants, and prooxidants have been evaluated on epimastigotes, and while antioxidants were able to reduce proliferation and increased metacyclogenesis, the oxidants and prooxidants had the opposite effect [44]. Those findings indicate that the redox state of the *T. cruzi* cell is important for cell growth and there must be a cell signaling pathway responsible of transducing the signal. Taken altogether, those results indicate that ROS has a direct effect on the parasite growth through a still unknown pathway of cell signaling.

### 1.4. Oxidative Stress in Chronic Phase Cardiomyopathy

Researchers disagree about the reasons for Chagas heart disease during the chronic phase, which develops in almost 30-40% of the patients and many years after the vector infects the host, but a strong connection between disease and the presence of the infective agent is still elusive. Chagas heart disease can occur after a cumulative and irreversible tissue destruction damage [30]; however, the reasons for the tissue damage are still not clear enough. More recently, the BENEFIT clinical trial treatment with benznidazole [59, 60], which was used at the chronic stage of Chagas disease, has revealed that once the damage is established, the progression of heart disease cannot be prevented even if a parasitological cure is achieved, as determined by the parasite clearance in the blood [61], indicating that the parasite presence is not responsible for disease progression. Those results suggest that other factors are involved in the heart disease progression produced by *T. cruzi* infection. However, those factors have not been fully identified yet.

Several works have succeeded to prevent [62, 63] and even reverse [57] the established functional heart disease by using an antioxidant therapy in mice and in an independent way of the therapy's effects on heart parasite burden. Those results suggest that Chagas cardiomyopathy is a ROS-dependent pathology, or at least ROS can contribute in a great extension to it [64]. In *T. cruzi*-infected cardiomyocyte cells, ROS is able to activate the NF-*κ*B activation pathway which induces the production of proinflammatory cytokines such as tumor necrosis factor (TNF) and IL-l*β* [65] that might lead to inflammatory responses able to affect heart function [66–68]. It is also possible that ROS might function as a funnel, in which several pathways of injury-causing factors, such as autoantibodies with adrenergic activities or others, can converge to disturb the myocardium physiology, most likely through the Calcium/Calmodulin Protein Kinase II-dependent phosphorylation pathway (CAMKII-like) and AMPK pathway of the host cells [69–71]. A successful work in the treatment of experimental Chagas disease at the chronic stage was done by Vilar-Pereira and colleagues using resveratrol [72]. This is a multitarget drug with cardioprotective properties exerted through NRF2, AMPK, SIRT1, SIRT3, and PGC1*α* activation [73, 74]. They infected mice with a lineage I *T. cruzi* strain and performed individual electrocardiography (ECG) and echocardiography studies before starting the treatment with the antioxidant resveratrol (at 60 days postinfection) and after another treatment was given at 90 days postinfection. At 90 days postinfection, those infected mice which were treated with resveratrol presented faster heart rate and shorter P wave duration, PR, and QT intervals when compared to infected mice treated without treatment [72]. Also, the treatment with resveratrol shortened P wave duration, PR, and QTc intervals, and increased individual heart rates during 60-90 days postinfection, and those benefits were not seen in infected mice without treatment. The resveratrol treatment activates the AMPK pathway and reduces parasite burden as well as the oxidative stress. Those results indicate that despite all tissue damage, there is a relevant physiological dysfunction in chronic Chagas heart disease which can be eventually reversed and improve cardiac function. This observation is important for the treatment of Chagas disease at the chronic stage.

Also, activation of the AMPK pathway or reducing ROS had similar beneficial effects as resveratrol on heart function during chronic Chagas disease. Metformin is an AMPK activator and has cardioprotective effects and also increases the expression of antioxidant enzymes such as mitochondrial SOD2, while tempol is a drug that neutralizes ROS, since it is a SOD-mimetic drug. The results of the treatments were similar to those with resveratrol, since the treated infected mice showed decreased PR and QTc intervals and also increased heart rates compared to infected mice without treatment [72]. However, metformin or tempol does not reduce parasite burden and these results indicate they do not act in a similar way as resveratrol. Taken altogether, these results suggest that reducing ROS and activating the AMPK pathway are sufficient to improve heart function in chronic Chagas disease even in the presence of the parasite.

In patients with Chagas disease, the activities of the mitochondrial respiratory complexes are depressed, a phenomenon associated with increased ROS production, which is correlated with a mitochondrial dysfunction [62]. Also, there is a high production of superoxide and H_2_O_2_ in the mitochondria of cardiomyocyte cells of mice chronically infected with *T. cruzi* [63]. This process is the result of electron leakage at the respiratory chain [64]. On the other hand, the treatment of the infected mice with the ROS-scavenger antioxidant PBN improves respiratory chain function and reduces electron leakage in the myocardium of those *T. cruzi*-infected mice [63]. Those results suggest that ROS production and mitochondrial dysfunction are intertwined in a positive feedback loop in this disease. Mice subjected to an acute *T. cruzi* infection produce a severe myocarditis which is characterized by inflammatory infiltrates containing macrophages, neutrophils, CD8 T cells, and tissue oxidative adducts. All of those effects can be prevented, or at least diminished, in the infected mice by the treatment with the NADPHox (NOS) inhibitor apocynin from day 0 of the infection [40]; however, the treatment increased blood and tissue parasite burden. The treatment decreases ROS produced by NADPHox, cytokine production, and T cell proliferation. When the treatment was extended to the chronic phase, no increase was observed in hypertrophy markers (increased cardiomyocyte cell size), fibrosis, or heart weight [40]. It was concluded from these studies that ROS produced by the NADPHox is a critical regulator of the splenic response such as cytokines, T cell, and phagocytic responses, which result in cardiac remodeling in mice with experimental Chagas disease at the chronic phase [40].

**Figure 2:**
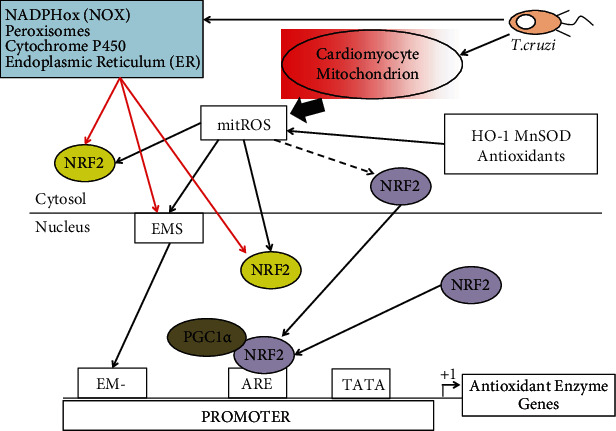
A model to explain the effect of increased mitochondrial ROS production on the antioxidant defenses of the cardiomyocytes. The main endogenous ROS sources are indicated. Also, exogenous ROS sources such as UV light, ionizing radiation, environmental agents, and drugs can contribute to ROS production. Mitochondrial ROS (mitROS) can impede the translocation to the nucleus of NRF2 and in addition can inactivate it both in the cytosol and nucleus (yellow NRF2) inhibiting the expression of the antioxidant enzyme genes, which can produce damage on the cell. On the other hand, antioxidant enzymes and antioxidants can neutralize the ROS effects on the NRF2 transcription factor (broken arrow) and this can translocate to the nucleus and activates the expression of antioxidant enzyme genes, leading to overcome the ROS effects. Additionally, ROS can activate an epigenetic marking system (EMS) in order to make a negative epigenetic mark (EM-) on the gene promoters of antioxidant enzymes, which leads to inhibition of the expression of those genes.

An imbalance between antioxidant and oxidant factors is considered the main cause of Chagas disease progression. As discussed earlier, immune cells produce ROS, RSN, proinflammatory cytokines, and peroxynitrite (ONOO-), which is a potent cytotoxic agent against parasites; however, it can also damage host cells. Cardiomyocytes also respond to *T. cruzi* infection by producing ROS, and several studies have shown that mitochondrion is the main source of ROS (mitochondrial ROS) in those infected cells [67, 68]. Increased mitochondrial ROS production is the result of loss of the structural integrity of the mitochondrial membrane and an effect on the membrane potential [67]. Altered mitochondrial function has been shown in *T. cruzi*-infected cardiomyocytes and in the myocardium of chronically infected animals [69, 75]. Studies in mice have shown that mitochondrial alterations and increased mitochondrial ROS are present in the chronic stage of infection [76, 77]. On the other hand, the increased ROS production during Chagas disease might cause the loss of key antioxidant enzymes of the host. It has been demonstrated that increased production of mitochondrial ROS inhibits the expression and activity of mitochondrial antioxidant enzyme MnSOD and the cytosolic GPx activity and also decreases the content of GSH in the myocardium of chronically infected animals and in Chagas patients [70]. As mentioned earlier, NRF2 is a known transcription factor that controls the gene expression of key antioxidant enzymes in cells under oxidative stress conditions [71]. Activation of NRF2 activity by CoPP induces HO-1 heme oxygenase, which reduces *T. cruzi* parasitemia and tissue parasitism; conversely, HO-1 inhibition increases *T. cruzi* in the blood [71]. Investigations on the role of mitochondrial ROS produced by *T. cruzi* infection show an improvement of the mitochondrial ROS scavenging capacity of infected mice by overexpressing the manganese superoxide dismutase (MnSOD) [78, 79]. In those studies, overexpression of MnSOD preserved the NRF2 transcriptional activity and binding to antioxidant response elements (ARE) on the promoter genes of antioxidant enzymes and also the expression of several enzymes of the antioxidant system, such as HO-1, gamma-glutamyl cysteine synthase (yGCS), and glutathione S transferase (GST). More importantly, the heart structure and function of those infected overexpressing transgenic mice was preserved [78]. Since *T. cruzi* infection in wild-type mice results in a ROS-dependent decrease of the NRF2 transcription factor activity indicates that preserving the NRF2 pathway will arrest the mitochondrial and cardiac oxidative damage in Chagas disease [78, 79]. These authors also considered that activation of other pathways, which have cardioprotective capacities in ROS-dependent cardiomyopathies, such as diabetes, could potentially restore mitochondrial functions, oxidative phosphorylation (OXPHOS) capacity, mitochondrial biogenesis, and all processes which are impaired in the myocardium of chronically infected mice [80]. For example, the sirtuin 1 (SIRT1) pathway can be activated in response to fasting as stressor and is able to activate several cell functions by deacetylating key cellular proteins, and it is connected to the energy-sensing AMP protein kinase pathway (AMPK) [81]. These two pathways seem to be coordinated with each other and can activate key proteins such as peroxisome proliferator-activated receptor gamma coactivator 1-alpha (PGC1*α*) [80]. PGC1*α* is a transcriptional coactivator that regulates mitochondrial biogenesis, energy expenditure, OXPHOS, and antioxidant defenses [81]. The authors were able to treat *T. cruzi*-infected mice during the late acute stage with the SIRT1 agonist SRT1720 and assessed heart mechanical function during chronic infection [80]. The SIRT1 activity in heart cells is decreased during the infection; however, resveratrol or SIRT1720 agonist treatment improved its activity along with left ventricular function, although it had no effects on cardiac remodeling or interstitial fibrosis [80]. The coactivator PGC1*α* was highly acetylated, but the agonist treatment did not improve the compromised mitochondrial biogenesis or the expression of mitochondrial genes. The SRT1720 therapy resulted in a decrease in H_2_O_2_ production, proinflammatory cytokine production, and cell infiltrate in the myocardium with Chagas disease [80]. The authors associated those benefits to the inhibition of the NF*κ*B transcriptional activity by a SIRT1-dependent mechanism [80]. Those results indicate that chronic cardiomyopathy in Chagas disease is a pathology that depends on ROS production. More importantly, it should be noted that the authors were able to intervene during the development of the disease and achieved long-term prevention, although they were not able to revert it. Taken altogether, those studies indicate that there is an exhaustion of the host antioxidant system during Chagas disease progression due to the increased mitochondrial ROS production and this can result in a persistent mitochondrial dysfunction and ROS production in cardiomyocytes [70, 76, 82]. The persisting mitochondrial ROS production can lead to the expression of fibrotic genes and this might contribute to the chronic cardiomyopathy. ROS also produces an effect on the antioxidant and immune response which could lead to a constant inflammation and the damage of proteins, lipids, and DNA which are characteristics of Chagas disease. It is also possible that mitochondrial ROS could signal long-lasting epigenetic modifications that keep an altered response in cardiomyocytes. All those observations are summarized in Figure 2.

The accumulated evidence suggests that oxidative stress is an important pathogenic factor in Chagas cardiomyopathy. The inhibition of oxidative stress seems to be critical for both improving and preventing heart dysfunction from developing in individuals with Chagas disease. One of the first antioxidants tested as a therapy for Chagas disease was vitamin E and it attenuates oxidative damage of the heart and in conjunction with benznidazole is able to prevent heart oxidative damage [83]. Selenium is already being tested as an antioxidant therapy in Chagas disease [84, 85]. It is expected that soon antioxidants acting through the NRF2 and/or SIRT1 pathways can be tested in clinical trials. All these results are pointing to a physiological heart dysfunction, caused mainly by ROS in individuals with Chagas disease, which can be treated and reversed with the appropriate antioxidants, AMPK activators, and SIRT agonists, despite the interference with parasite burden or cardiac hypertrophy. Some of those drugs are being tested experimentally in mice, and we hope soon those drugs can be tested in clinical trials to evaluate their use in patients with Chagas disease [86]. More importantly, those drugs and new research could open new frontiers in the treatment and in the development of specific drugs to treat and cure Chagas disease.

### 1.5. DNA Metabolism under Oxidative Stress in *T. cruzi*

When the production of ROS exceeds the antioxidant defense capacity, lesions on the DNA are formed, which can be lethal unless they are repaired before replication occurs. As we have discussed, *T. cruzi* is an intracellular parasite and the host-invaded cells can produce high amounts of H_2_O_2_ in response to the infection. H_2_O_2_ can trespass membranes and produce damage of parasite and host molecules. *T. cruzi* must to be able to prevent oxidant formation and repair an eventual damage of its own DNA, in order to survive inside the host cells. *T. cruzi* has evolved a series of evolutionary adaptation mechanisms to adapt to the harsh oxidative conditions inside the host cells. In this section, we will focus on the mechanisms by which *T. cruzi* can deal with DNA lesions produced by ROS. However, we must also remember that host-infected cells can be able to activate DNA repair pathways in response to the high ROS produced by the infection which elicits DNA damage.

The DNA is a key target of ROS into the cells, since it represents the template molecule for replication and transcription. Another ROS targets are proteins, membrane phospholipid, and RNAs; however, those are in many copies and in a constant turnover and might not be as important as the DNA. ROS attacks the nitrogenated bases and the sugar-phosphate backbone of DNA producing single- and double-strand breaks (SSB and DSB, respectively), base modifications, apurinic/apyrimidinic (AP) sites, and DNA-protein crosslinks [72, 81, 82]. While DBS are lethal if left unrepaired, oxidized bases might be mutagenic, cytotoxic, or even both of them. One of the most prevalent and mutagenic lesions is 8-oxoguanine (8-oxoG) [87–89]. In eukaryotic cells, different kinds of lesions can be produced in more than one repair DNA system that has evolved to deal with oxidative damage and they form an intricate network that many times can have a functional overlap between them. The DNA breaks are repaired by the recombination pathways, which can proceed through nonhomologous end joining (NHE) or homologous recombination (HR) [87–89]. On the other hand, modified bases are primarily removed by DNA glycosylases, which can produce abasic sites that are repaired by the base excision repair (BER) pathway [87–89]. It has been also suggested that the nucleotide incision repair (NIR) pathway could serve as a backup for BER in the removal of cytosine-derived lesions [87–89]. The nucleotide excision repair (NER) system has been also described as an alternative system for BER to deal with oxidative lesions such as thymine glycol and abasic sites, and the NER enzymes can be regulated by oxidative stress [72, 81, 82]. Moreover, the mismatch repair (MMR) system can control the levels of 8-oxoG in DNA and its role is in the recognition of oxidative lesions which is generally accepted by the researchers in the field [87–89]. The DNA repair process in trypanosomatids has not been extensively studied; however, its understanding has advanced by the publication of the genome sequences from several of them, including *T. cruzi* [90, 91]. The majority of the enzyme-encoding genes involved in the aforementioned DNA repair processes are present in *T. cruzi* [90, 91].

Those DNA lesions in which the bases are modified by the addition or loss of a small chemical group are repaired by the BER system [87–89]. The classical BER repair pathway begins with the recognition of modified by the DNA glycosylases. Monofunctional DNA glycosylases remove the modified base producing an intact AP site, which is subsequently recognized and acted upon by AP endonucleases (such as APE1), which leaves a free 3′-OH and a 5′-deoxyribose phosphate terminus. However, most of the DNA glycosylases involved in the DNA repair of oxidative damage are bifunctional and have an associated AP lyase enzyme activity [87–89]. After the recognition of the lesion, the enzyme can remove the modified base and cleave the DNA at 3′ side from the AP site produced. This event produces a baseless sugar retained on the 3′ end, which is subsequently removed by another endonuclease, named APE1. No 5′-deoxyribose phosphate flap is produced in this case [87–89]. After the base is removed, short patch (SP-BER, single-nucleotide replacement) or long patch (LP-BER, multinucleotide replacement) repair might proceed. All these DNA repair mechanisms end up with the actions of DNA polymerases and ligases to seal the gap. Both processes SP-BER and LP-BER involve distinct sets of enzymes and accessory proteins. In SP-BER for example, DNA pol *β* synthesizes the missing nucleotide extending from the free 3′-OH terminal [87–89]. When a 5′-deoxyribose phosphate flap is present, DNA pol *β* uses its lyase activity to remove it. The gap is sealed by DNA ligase III and stimulated by XRCC1 [87–89]. In LP-BER, on the other hand, replicative DNA polymerases, such as pol*δ* and pol*ε*, are the enzymes in charge of the replication/elongation step and the sealing is carried out by DNA ligase I [87–89]. Sequences encoding essential DNA repair enzymes such as replication factor C, PCNA, APE1/APE2, FEN-1, ligase I, PARP1, DNA pol*β*, DNA pol*δ* and DNA pol*ε*, and several other DNA polymerases have been identified in the *T*. *cruzi* genome [90, 91]. Also, a gene encoding a DNA glycosylase has been found in *T. cruzi* genome [92]. However, genes encoding DNA ligase III and XRCC1 have not been found, which implies that other enzymes are responsible for the ligation step in SP-BER. Evidence indicates that treatment of *T. cruzi* epimastigotes with high concentrations of ROS can damage the nuclear and kinetoplast DNA and this damage might be partially repaired by the BER pathway [93]. An inhibitor of the BER pathway repair system (methoxyamine) augments the negative effect of ROS on cell viability, suggesting the involvement of this pathway [93].

**Figure 3:**
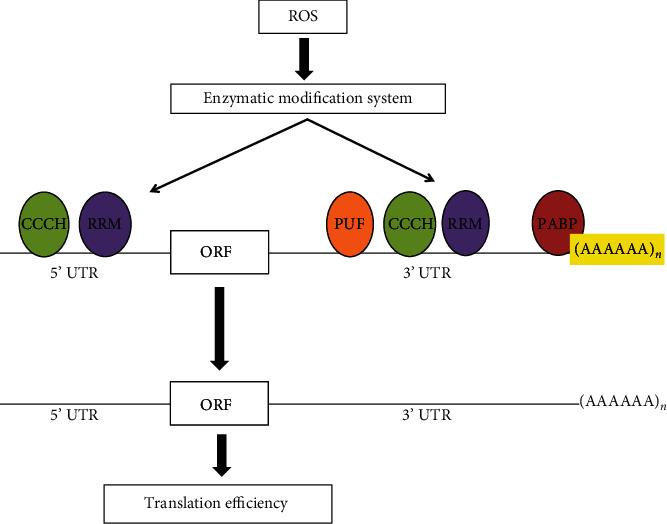
Regulation of Tcpol*β* expression at the posttranscriptional level. The Tcpol*β* mRNA is bound at the 5′ UTR, 3′ UTR, and poly A tail by different RNA binding proteins, which mainly contains the RNA recognition motif (RRM), Pumilio/Fem-3 mRNA domain (PUF), the zinc finger CCCH domain, and the poly A binding protein (PABP), which can act from splicing to RNA function and turnover. The Tcpol*β* mRNA can be bound by any of those proteins and increase the splicing rate and/or decrease the translation efficiency of the mRNA. A signal provided by ROS could act on an enzymatic modifying system, which in turn acts on the bound factors, causing the release from the mRNA to increase translation efficiency. The PUF family binds to the 3′ UTRs, while CCCH domain and the RRH domain binds to both 5′ and 3′ UTRs. Those proteins bind to specific sequences on the UTRs.

Interestingly, *T. cruzi* possesses two DNA pol *β* homologues: Tcpol*β* and Tcpol*β*-PAK [85]. Both enzymes are localized in the parasite kinetoplast and they present intrinsic 5′-dexoxyribose phosphate lyase activity *in vitro*, the same as the mammalian counterparts [94]. This implies that in *T. cruzi*, in the absence of a nuclear pol*β* to carry out SP-BER, LP-BER would be most likely the sole BER pathway available in the nucleus. It is noteworthy mentioning that recombinant Tcpol*β*-PAK is able to perform DNA synthesis over 8-oxoG *in vitro* suggesting its involvement in a pathway named translesion synthesis (TLS) in mitochondrial DNA [94]. This pathway will be discussed in more detail later. On the other hand, Tcpol*β* does not possess this ability; however, its overexpression enhances the survival of *T. cruzi* strains to H_2_O_2_ [94]. Moreover, recombinant Tcpol*β* requires additional cellular proteins to be able to repair *in vitro* short gaps of DNA, suggesting that in vivo is part of a multiprotein repair system [95]. Whether or not Tcpol*β* can perform mitochondrial TLS *in vivo* remains to be investigated. There is evidence for a role of Tcpol*β* in dealing with oxidative lesions of the kinetoplast DNA, most likely through the BER mechanism of repair. Indeed, when epimastigote cells are treated with H_2_O_2_, a new focus for Tcpol*β* localization is detected, besides the two Tcpol*β* foci, which are localized on kinetoplast DNA antipodal sites in the absence of treatment, suggesting that it could correspond to a DNA repair site [94]. The parasites overexpressing Tcpol*β* displayed reduced levels of 8-oxoG in the kinetoplast DNA when compared to normal cells [94]. Furthermore, we have demonstrated that *T. cruzi* epimastigote and trypomastigote cells can respond to H_2_O_2_ treatment expressing higher levels of Tcpol*β* compared to untreated cells [87]. Tcpol*β* can be found in two forms in *T. cruzi* cell extracts. The high form is heavily phosphorylated (H), while the low form is unphosphorylated (L). Interestingly, the high form is more active in *in vitro* DNA synthesis [96]. The levels of the phosphorylated active form of the enzyme are higher in H_2_O_2_-treated cells compared with untreated cells, suggesting that this activity is involved in repair of the kinetoplast DNA and perhaps is also responsible of the kinetoplast DNA replication [96]. A mechanistic model to explain the overexpression of Tcpol*β* is outlined in Figure 3 and it can be observed the different protein families involved in the posttranscriptional expression of the enzyme. Tcpol*β* can be crosslinked to kinetoplast DNA, but no to nuclear DNA, indicating that the enzyme is always in contact with kinetoplast DNA [96]. We believe that several protein kinases are involved in the phosphorylation of Tcpol*β* and those kinases are involved in the signal transduction pathway triggered by the H_2_O_2_ treatment. Perhaps, a similar pathway operates to transduce the positive growth signal triggered by ROS on *T. cruzi*. Those protein kinases can act either by phosphorylating Tcpol*β* and/or by modifying proteins that can be bound to the Tcpol*β* mRNA in order to make it a better template for protein translation, thus increasing the activity and the amount of the enzyme. Most likely, other proteins and DNA polymerases involved in the process of DNA repair can be positively modulated by H_2_O_2_ treatment, but those experiments will wait until specific antibodies against them can be developed. Another mitochondrial located DNA polymerase which is able to perform TLS *in vitro* is Tcpol*κ*, which can bypass 8-oxoG lesions *in vitro*, and its overexpression together with Tcpol*β* and Tcpol*β*-PAK in *T. cruzi* cells augments parasite survival against benznidazole [97].

Since the trypanosomatids possess a single mitochondrion, it is possible that they need a powerful system for DNA repair, because the mitochondria are important source of oxidative stress and many mitochondrial-encoded proteins are important for parasite survival. Also, the efficient kinetoplast DNA replication is essential for parasite duplication, since any blocks in the replication fork could affect the parasite viability. Therefore, the mitochondrial BER activity given by Tcpol*β* and TLS activity of Tcpol*β*-PAK and Tcpol*κ* are essential for the parasite and hence allow the progression of kinetoplast DNA replication even when oxidative lesions are present.

As mentioned earlier, in addition to BER and the repair systems mentioned earlier, a tolerance pathway, called TLS, has evolved from bacteria to humans. This system contains DNA polymerases able to replicate the DNA across several DNA lesions, such as 8-oxoG and thymine glycol. This TLS system is able to ensure genome integrity, but generates increased mutations, since the TLS polymerases have lower fidelity than those involved in DNA replication. Several genes encoding TLS DNA polymerases are present in the *T. cruzi* genome including pol*β*, pol*η*, and pol*κ*, indicating TLS can be operative in *T. cruzi* [87–89, 97, 98]. On the other hand, it has been shown that Tcpol*η*, a nuclear located DNA polymerase, has TLS activity *in vitro* and its overexpression in *T. cruzi* cells leads to higher parasite survival in the presence of H_2_O_2_ [98]. This DNA polymerase can bypass cyclobutene pyrimidine dimers and also can bypass 8-oxoG lesions. Its ability to confer resistance to oxidative lesions might be associated with the ability to bypass 8-oxoG lesions in the DNA. This suggests that Tcpol*η* has a role in TLS of nuclear DNA oxidative damage.

**Figure 4:**
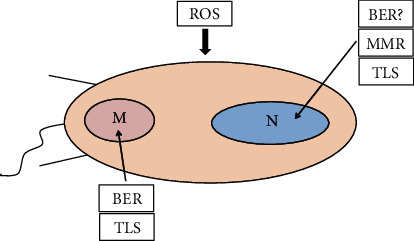
DNA repair mechanisms under oxidative stress. *T. cruzi* has several mechanisms to repair the damage caused by ROS. To repair, the nuclear DNA possesses the mismatch repair system (MMR), the translesion synthesis repair system (TLS), and likely a base excision-like repair system (BER-like). The kinetoplast DNA is repaired by a base excision repair system (BER, Tcpol*β*, and Tcpol*β*-PAK) and a TLS repair system. See text for details.

Despite the fact that BER is the main repair pathway to repair the oxidized base 8-oxoG, another repair pathway might also operate in *T. cruzi* to repair this lesion in the nuclear DNA. In higher eukaryotes, it is involved in the repair of oxidative lesions in the nuclear DNA and it is named mismatch repair (MMR) system. The proteins able to recognize the mismatches in the DNA are a dimer of MSH2-MSH6, which is the *E. coli* MutS system counterpart. Both MSH2 and MSH6 genes are present in *T. cruzi*, and different isoforms of MSH2 exist [99, 100]. Moreover, *T. cruzi* strains, in which one of the chromosomal copies of MSH2 was deleted, are more sensitive to H_2_O_2_ treatment than the wild-type cells [100]. On the contrary, *T. cruzi* strains lacking both copies of MSH2 genes are more resistant to H_2_O_2_ treatment than the wild-type parasites [100]. Although MSH2 might have a role dealing with oxidative lesions of the DNA, it seems to be that the MMR is not directly involved in the repair of oxidative damage and perhaps the MSH proteins could be involved in other signaling pathways, such as the signaling per se of oxidative damage to other repair systems. The finding that basal 8-oxoG levels are higher in H_2_O_2_ T*. cruzi* hybrid strains (lineage II) compared to *T. cruzi* of the lineage I could corroborate the hypothesis that *T. cruzi* from the lineage I are more resistant to H_2_O_2_ than those hybrid strains. This means that higher 8-oxiG levels in *T. cruzi* hybrid strains would implicate in the recruitment of high numbers of MSH2 protein to deal with the lesions, thus affecting the MMR pathway efficiency under stressful conditions, such as treatment with genotoxic agents [101]. An overview of the *T. cruzi* DNA repair system location is given in Figure 4. BER repair system has not been found in the nucleus; however, a BER-like repair system operating at the nucleus cannot be ruled out yet.

In general, the role of the enzymes involved in the oxidative stress response can be demonstrated upon the overexpression of those enzymes and the increased tolerance that the parasites show upon ROS exposure. For example, the role of BER in the oxidative stress response can be observed in parasites overexpressing the APE1 endonuclease, which show an increased tolerance to persistent ROS exposure [102]. On the contrary, parasites treated with a BER pathway inhibitor methoxyamine have a diminished cell viability in the presence of high concentrations of ROS, which can cause nuclear and kinetoplast DNA damage [93].

## 2. Concluding Remarks and Future Directions

It has become clear that ROS produced by *T. cruzi*-infected cells have several functions on the host-pathogen interactions. ROS produces a positive signal on *T. cruzi*, which induces cell growth. On the other hand, ROS is able to activate a cell signal pathway in the host cell, leading to proinflammatory cytokine production. Moreover, ROS can produce a signal, which induces chronic heart tissue damage in the chagasic patient, even several years (decades) after the infection was produced. It can be concluded that *T. cruzi* possesses powerful antioxidant defense systems, which can deal with ROS produced by the host cell. The detoxification systems use thiol compounds and several enzymes to detoxify the ROS molecules produced mainly inside the mitochondria of the host-infected cell. To protect the DNA against ROS-produced oxidative damage, *T. cruzi* has several DNA repair systems including BER, TLS, MMR, and perhaps another unidentified yet DNA repair systems.

Seems to be that parasite burden is not necessarily responsible for chronic heart damage and classical antichagasic drugs no longer function to treat the disease at the chronic stage. Thus, there is a need to develop new drugs to treat the disease and antioxidants, drugs that can activate the expression of the host antioxidant enzymes or that can inhibit cytokine, and ROS productions are the most promising to treat the Chagas disease at the chronic stage. Also, the possibility of using combined therapies must be considered.

Although there are plenty of information on the effects among host-pathogen interactions during *T. cruzi* infection, several issues on the molecular mechanisms by which ROS produces beneficial effects on the pathogen and harmful effects on the host must be explained. Although it is quite difficult to envision a mechanism to explain the persistent signal that ROS produces on the host leading to accumulative progressive heart tissue damage, even decades after the initial infection and in the absence of the pathogen. Also, it is necessary to explain whether or not ROS produced by the host acts directly on the parasite by producing a cell signal to enhance its growth.

**Figure 5:**
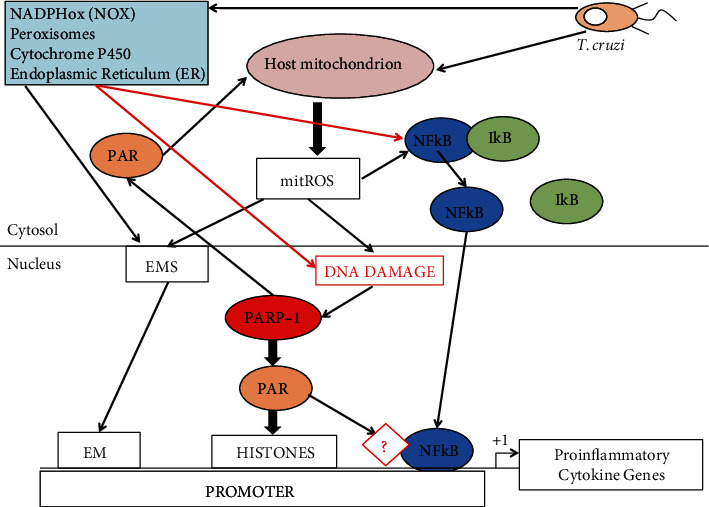
A model to explain the tissue damage in the host heart at the chronic stage of Chagas disease. The parasite inside the cells induces ROS production by the mitochondria of the host cell and also by the NADPHox. The ROS signal produces nuclear DNA damage, PARP activation, epigenetic marking system (EMS) activation, and NF*κ*B translocation by dissociation of I*κ*B. PARP-1 (poly ADP-ribose polymerase) produces poly ADP-ribose (PAR) polymers, which help the binding of NF*κ*B on the gene promoter and produce epigenetic marks (EM) on the promoter histones, which together with the binding of NF*κ*B can activate proinflammatory cytokine gene expression. Most likely another factor (?) is activated by PAR and helps NF*κ*B to bind to the gene promoter. Also, an EM can be done on the gene promoter by the EMS keeping the proinflammatory cytokine genes active, leading to cardiac remodeling. On the other hand, PAR from the nucleus can travel to the cytosol and provides a signal to the mitochondrion to keep producing ROS. In addition, PARP-1 activity facilitates DNA repair. This model is complementary to that presented in Figure 2, since inactivation of the NRF2 axis can cause damage on the cell.

We could put forward the following hypothesis to explain those observations:

1. At the beginning of the infection, the ROS produced in response to *T. cruzi* infection can elicit a signal transduction pathway on the pathogen in order to be induced to proliferate. That signal could be sensed and transduced trough the MAPK pathway, since the *T. cruzi* genome contains several genes encoding potential MAPKs and in higher eukaryotes those are known to play important roles in cell signaling regulating key processes such as proliferation and stress response. We could image that a MAPK kinase senses and transduces the ROS signal phosphorylating and also by increasing the production of key enzymes and proteins, such as those involved cell division, transcription, DNA replication, DNA repair, and others. It must be noted that those MAPKs should act at the translational/modification level, since transcriptional regulation has not been described in *T. cruzi*
2. At the chronic stage of the infection, *T. cruzi*-infected cardiomyocytes could produce a long-lasting mark on its own molecules, which persists for a long time. We could imagine that an epigenetic mark could be made on the genome DNA, specifically on those gene promoters, which encode proinflammatory cytokine genes in order to maintain the genes on. Alternatively, an epigenetic mark could be made on gene promoters encoding transcription factors, which control the expression of antioxidant enzymes, in order to keep these genes off. For example, that mark could be a *T. cruzi*-induced methylation on the gene promoters, which can regulate the activity of these gene promoters and therefore the expression of those genes. This hypothesis is outlined in Figure 5

The identification of MAPKs, transducing the ROS signal, might be good candidates for new drug development against *T. cruzi* in order to obtain a high arsenal of drugs to treat the Chagas disease, since there is a very limited number of drugs to treat the disease, and they are rather toxic. Assuming that a protein key kinase or phosphatase crucial to regulate a biological process in *T. cruzi*, however, is different from a human counterpart, a specific inhibitor could be found or designed, thus affecting mainly the parasite protein without harming the host. With the availability of the *T. cruzi* genome, a detailed biochemical analysis of kinases and phosphatases could be done, to improve the knowledge of their characteristics and to be used to develop new therapeutic agents against Chagas disease. Studies can be designed to identify the long-lasting signal that persists in the cardiomyocytes, even in the absence of parasite burden, and additionally, the positive ROS-provided signal to the pathogens must be identified. It is possible that with the new tools of genomics, transcriptomics and proteomics those alterations and signals might be identified in a near future.

## Abbreviations

ROS: Reactive oxygen species
RNS: Reactive nitrogen species
TR: Trypanothione
T(SH_2_): Dithiol trypanothione
TcTXN: Tryparedoxin
TcTS: Trypanothione synthetase
TcTR: Trypanothione reductase
TcAPX: Ascorbate-dependent hemoperoxidase
TcMPX/TcCPX: Tryparedoxin peroxidase
GPX-I: Glutathione-dependent peroxidase I
FeSOD-A: Iron-dependent superoxide dismutase
MnSOD: Manganese-dependent superoxide dismutase
GPX-II: Glutathione-dependent peroxidase II
NADPHox: NADPH oxidase
IFN: Interferon
Tcpol: *T. cruzi* polymerase
BER: Base excision repair
MMR: Mismatch repair
TLS: Translesion synthesis
NF*κ*B: Nuclear factor kappa-light-chain-enhancer of activated B cells.

## References

[1] Echeverria L. E., Morillo C. A. (2019). American Trypanosomiasis (Chagas Disease). *Infectious Disease Clinics of North America*.

[2] Lidani K. C. F., Andrade F. A., Bavia L. (2019). Chagas Disease: From Discovery to a Worldwide Health Problem. *Frontiers in Public Health*.

[3] Liu Q., Chen J., Zhou X. N. (2020). Preparedness for Chagas disease spreading worldwide. *Infectious Diseases of Poverty*.

[4] Burleigh B. A. (2019). Metabolic interplay and flexibility in the intracellularTrypanosoma cruzi‐host cell interaction. *The FASEB Journal*.

[5] Suo X., Wu Z., Lillehoj H., Tuo W. (2020). Editorial: Immunoparasitology: a unique interplay between host and pathogen. *Frontiers in Immunology*.

[6] Prata A. (2001). Clinical and epidemiological aspects of Chagas disease. *The Lancet Infectious Diseases*.

[7] Kratz J. M. (2019). Drug discovery for chagas disease: A viewpoint. *Acta Tropica*.

[8] Peacock C. S. (2018). The practical implications of comparative kinetoplastid genomics. *Comparative Genomics and Proteomics in Drug Discovery*.

[9] Jackson A. P., Otto T. D., Aslett M. (2016). Kinetoplastid phylogenomics reveals the evolutionary innovations associated with the origins of parasitism. *Current Biology*.

[10] Dejung M., Subota I., Bucerius F. (2016). Quantitative Proteomics Uncovers Novel Factors Involved in Developmental Differentiation of Trypanosoma brucei. *PLOS Pathogens*.

[11] Gonçalves C. S., Ávila A. R., de Souza W., Motta M. C. M., Cavalcanti D. P. (2018). Revisiting the Trypanosoma cruzi metacyclogenesis: morphological and ultrastructural analyses during cell differentiation. *Parasites & Vectors*.

[12] Piacenza L., Alvarez M. N., Peluffo G., Radi R. (2009). Fighting the oxidative assault: the Trypanosoma cruzi journey to infection. *Current Opinion in Microbiology*.

[13] Piacenza L., Peluffo G., Alvarez M. N., Martínez A., Radi R. (2013). Trypanosoma cruzi antioxidant enzymes as virulence factors in Chagas disease. *Antioxidants & Redox Signaling*.

[14] Machado-Silva A., Cerqueira P. G., Grazielle-Silva V. (2016). How Trypanosoma cruzi deals with oxidative stress: antioxidant defence and DNA repair pathways. *Mutation Research/Reviews in Mutation Research*.

[15] Piacenza L., Zago M. P., Peluffo G., Alvarez M. N., Basombrio M. A., Radi R. (2009). Enzymes of the antioxidant network as novel determiners of Trypanosoma cruzi virulence. *International Journal for Parasitology*.

[16] Trujillo M., Budde H., Piñeyro M. D. (2004). Trypanosoma bruceiandTrypanosoma cruziTryparedoxin peroxidases catalytically detoxify peroxynitrite via oxidation of fast reacting thiols. *Journal of Biological Chemistry*.

[17] Freire A. C. G., Alves C. L., Goes G. R. (2017). Catalase expression impairs oxidative stress-mediated signalling in Trypanosoma cruzi. *Parasitology*.

[18] Piñeyro M. D., Parodi-Talice A., Portela M., Arias D. G., Guerrero S. A., Robello C. (2011). Molecular characterization and interactome analysis of Trypanosoma cruzi tryparedoxin 1. *Journal of Proteomics*.

[19] Arias D. G., Piñeyro M. D., Iglesias A. A., Guerrero S. A., Robello C. (2015). Molecular characterization and interactome analysis of Trypanosoma cruzi tryparedoxin II. *Journal of Proteomics*.

[20] Wilkinson S. R., Temperton N. J., Mondragon A., Kelly J. M. (2000). Distinct mitochondrial and cytosolic enzymes mediate trypanothione-dependent peroxide metabolism inTrypanosoma cruzi. *Journal of Biological Chemistry*.

[21] Arias D. G., Marquez V. E., Chiribao M. L. (2013). Redox metabolism in Trypanosoma cruzi: functional characterization of tryparedoxins revisited. *Free Radical Biology and Medicine*.

[22] Piñeyro M. D., Arcari T., Robello C., Radi R., Trujillo M. (2011). Tryparedoxin peroxidases from Trypanosoma cruzi: High efficiency in the catalytic elimination of hydrogen peroxide and peroxynitrite. *Archives of Biochemistry and Biophysics*.

[23] Nogueira F. (2009). Molecular characterization of cytosolic and mitochondrial tryparedoxin peroxidase in Trypanosoma cruzi populations susceptible and resistant to benznidazole. *Parasitology Research*.

[24] Phan I. Q. H., Davies D. R., Moretti N. S. (2015). Iron superoxide dismutases in eukaryotic pathogens: new insights from Apicomplexa and Trypanosoma structures. *Acta Crystallographica Section F: Structural Biology Communications*.

[25] Piacenza L., Peluffo G., Alvarez M. N., Kelly J. M., Wilkinson S. R., Radi R. (2008). Peroxiredoxins play a major role in protecting Trypanosoma cruzi against macrophage-and endogenously-derived peroxynitrite. *Biochemical Journal*.

[26] Wilkinson S. (2002). Trypanosoma cruzi expresses a plant-like ascorbate-dependent hemoperoxidase localized to the endoplasmic reticulum. *Proceedings of the National Academy of Sciences*.

[27] Denicola A., Rubbo H., Rodriguez D., Radi R. (1993). Peroxynitrite-mediated cytotoxicity to Trypanosoma cruzi. *Archives of Biochemistry and Biophysics*.

[28] Alvarez M. N., Piacenza L., Irigoín F., Peluffo G., Radi R. (2004). Macrophage-derived peroxynitrite diffusion and toxicity to Trypanosoma cruzi. *Archives of Biochemistry and Biophysics*.

[29] Teixeira M. (2002). Chemokines, inflammation and Trypanosoma cruzi infection. *Trends in Parasitology*.

[30] Lopez M., Tanowitz H. B., Garg N. J. (2018). Pathogenesis of chronic Chagas disease: macrophages, mitochondria, and oxidative stress. *Current Clinical Microbiology Reports*.

[31] Paiva C. N., Medei E., Bozza M. T. (2018). ROS and Trypanosoma cruzi: fuel to infection, poison to the heart. *PLoS Pathogens*.

[32] Alvarez M. N., Peluffo G., Piacenza L., Radi R. (2011). Intraphagosomal peroxynitrite as a macrophage-derived cytotoxin against internalized trypanosoma cruzi consequences for oxidative killing and role of microbial peroxiredoxins in infectivity. *Journal of Biological Chemistry*.

[33] Mesías A. C., Garg N. J., Zago M. P. (2019). Redox balance keepers and possible cell functions managed by redox homeostasis in Trypanosoma cruzi. *Frontiers in Cellular and Infection Microbiology*.

[34] Koo S.-j., Chowdhury I. H., Szczesny B., Wan X., Garg N. J. (2016). Macrophages promote oxidative metabolism to drive nitric oxide generation in response to Trypanosoma cruzi. *Infection and Immunity*.

[35] Arantes R. M. E., Marche H. H. F., Bahia M. T., Cunha F. Q., Rossi M. A., Silva J. S. (2004). Interferon-*γ*-induced nitric oxide causes intrinsic intestinal denervation in Trypanosoma cruzi-infected mice. *The American Journal of Pathology*.

[36] Saeftel M., Fleischer B., Hoerauf A. (2001). Stage-dependent role of nitric oxide in control of Trypanosoma cruzi infection. *Infection and Immunity*.

[37] Santos-Miranda A., Joviano-Santos J. V., Ribeiro G. A. (2020). Reactive oxygen species and nitric oxide imbalances lead to in vivo and in vitro arrhythmogenic phenotype in acute phase of experimental Chagas disease. *PLoS Pathogens*.

[38] de Almeida Nogueira N. P., de Souza C. F., de Souza Saraiva F. M. (2011). Heme-induced ROS in Trypanosoma cruzi activates CaMKII-like that triggers epimastigote proliferation. One helpful effect of ROS. *PloS one*.

[39] Goes G. R., Rocha P. S., Diniz A. R. S., Aguiar P. H. N., Machado C. R., Vieira L. Q. (2016). Trypanosoma cruzi needs a signal provided by reactive oxygen species to infect macrophages. *PLoS Neglected Tropical Diseases*.

[40] Nogueira N. P., Saraiva F. M. S., Oliveira M. P. (2017). Heme modulates Trypanosoma cruzi bioenergetics inducing mitochondrial ROS production. *Free Radical Biology and Medicine*.

[41] Andrade L. O., Dias P. P. (2019). Role of ROS in T. cruzi intracellular development. *Oxidative Stress in Microbial Diseases*.

[42] da Silva Augusto L., Moretti N. S., Ramos T. C. P. (2015). A membrane-bound eIF2 alpha kinase located in endosomes is regulated by heme and controls differentiation and ROS levels in Trypanosoma cruzi. *PLoS Pathog*.

[43] Tanowitz H. B., Wen J.-j., Machado F. S., Desruisseaux M. S., Robello C., Garg N. J. (2016). Trypanosoma cruzi and Chagas disease: innate immunity, ROS, and cardiovascular system. *Vascular Responses to Pathogens*.

[44] Sánchez-Villamil J. P., Bautista-Niño P. K., Serrano N. C., Rincon M. Y., Garg N. J. (2020). Potential role of antioxidants as adjunctive therapy in Chagas disease. *Oxidative Medicine and Cellular Longevity*.

[45] Paiva C. N., Feijó D. F., Dutra F. F. (2012). Oxidative stress fuels Trypanosoma cruzi infection in mice. *The Journal of Clinical Investigation*.

[46] Silva R. C. M. C., Travassos L. H., Paiva C. N., Bozza M. T. (2020). Heme oxygenase-1 in protozoan infections: a tale of resistance and disease tolerance. *PLoS Pathogens*.

[47] Finzi J. (2004). Trypanosoma cruzi response to the oxidative stress generated by hydrogen peroxide. *Molecular and Biochemical Parasitology*.

[48] Schnapp A. (2002). Cruzipain induces both mucosal and systemic protection against Trypanosoma cruzi in mice. *Infection and Immunity*.

[49] Guiñazú N., Carrera-Silva E. A., Becerra M. C., Pellegrini A., Albesa I., Gea S. (2010). Induction of NADPH oxidase activity and reactive oxygen species production by a single Trypanosoma cruzi antigen. *International Journal for Parasitology*.

[50] Nagajyothi F., Zhao D., Weiss L. M., Tanowitz H. B. (2012). Curcumin treatment provides protection against Trypanosoma cruzi infection. *Parasitology Research*.

[51] Novaes R. D., Sartini M. V. P., Rodrigues J. P. F. (2016). Curcumin enhances the anti-Trypanosoma cruzi activity of benznidazole-based chemotherapy in acute experimental Chagas disease. *Antimicrobial Agents and Chemotherapy*.

[52] Brazão V., Colato R. P., Santello F. H. (2018). Effects of melatonin on thymic and oxidative stress dysfunctions during Trypanosoma cruzi infection. *Journal of Pineal Research*.

[53] Sabelli M., Montosi G., Garuti C. (2017). Human macrophage ferroportin biology and the basis for the ferroportin disease. *Hepatology*.

[54] Andrews N. W. (2012). Oxidative stress and intracellular infections: more iron to the fire. *Journal of Clinical Investigation*.

[55] Paes M. C. (2019). The journey of Trypanosoma cruzi under the redox baton. *Biology of Trypanosoma cruzi.*.

[56] Souza C. F., Carneiro A. B., Silveira A. B. (2009). Heme-induced Trypanosoma cruzi proliferation is mediated by CaM kinase II. *Biochemical and Biophysical Research Communications*.

[57] Mesquita I., Moreira D., Sampaio-Marques B. (2016). AMPK in pathogens. *AMP-Activated Protein Kinase*.

[58] Malvezzi A. M., Aricó M., Souza-Melo N. (2020). GCN2-like kinase modulates stress granule formation during nutritional stress in Trypanosoma cruzi. *Frontiers in Cellular and Infection Microbiology*.

[59] Poveda C., Fresno M., Gironès N. (2014). Cytokine profiling in Chagas disease: towards understanding the association with infecting Trypanosoma cruzi discrete typing units (a BENEFIT TRIAL sub-study). *PloS One*.

[60] Morillo C. (2015). Randomized trial of benznidazole for chronic Chagas’ cardiomyopathy. *New England Journal of Medicine*.

[61] Moreira O. (2013). Towards the establishment of a consensus real-time qPCR to monitor Trypanosoma cruzi parasitemia in patients with chronic Chagas disease cardiomyopathy: a substudy from the BENEFIT trial. *Acta Tropica*.

[62] Wen J.-j., Yachelini P. C., Sembaj A., Manzur R. E., Garg N. J. (2006). Increased oxidative stress is correlated with mitochondrial dysfunction in chagasic patients. *Free Radical Biology and Medicine*.

[63] Gupta S., Smith C., Auclair S., Delgadillo A. J., Garg N. J. (2015). Therapeutic efficacy of a subunit vaccine in controlling chronic Trypanosoma cruzi infection and chagas disease is enhanced by glutathione peroxidase over-expression. *PLoS One*.

[64] Wen J.-J., Garg N. (2004). Oxidative modification of mitochondrial respiratory complexes in response to the stress of Trypanosoma cruzi infection. *Free Radical Biology and Medicine*.

[65] Ba X., Gupta S., Davidson M., Garg N. J. (2010). Trypanosoma cruzi induces the reactive oxygen species-PARP-1-RelA pathway for up-regulation of cytokine expression in cardiomyocytes. *Journal of Biological Chemistry*.

[66] Gupta S., Dhiman M., Wen J.-j., Garg N. J. (2011). ROS signalling of inflammatory cytokines during Trypanosoma cruzi infection. *Advances in Parasitology*.

[67] Gupta S., Bhatia V., Wen J.-j., Wu Y., Huang M.-H., Garg N. J. (2009). Trypanosoma cruzi infection disturbs mitochondrial membrane potential and ROS production rate in cardiomyocytes. *Free Radical Biology and Medicine*.

[68] Trocoli Torrecilhas A. C., Tonelli R. R., Pavanelli W. R. (2009). Trypanosoma cruzi: parasite shed vesicles increase heart parasitism and generate an intense inflammatory response. *Microbes and Infection*.

[69] Wen J.-J., Vyatkina G., Garg N. (2004). Oxidative damage during chagasic cardiomyopathy development: role of mitochondrial oxidant release and inefficient antioxidant defense. *Free Radical Biology and Medicine*.

[70] Beckendorf J., van den Hoogenhof M. M. G., Backs J. (2018). Physiological and unappreciated roles of CaMKII in the heart. *Basic Research in Cardiology*.

[71] Paunkov A., Chartoumpekis D. V., Ziros P. G., Sykiotis G. P. (2019). A bibliometric review of the Keap1/Nrf2 pathway and its related antioxidant compounds. *Antioxidants*.

[72] Vilar-Pereira G., Carneiro V. C., Mata-Santos H. (2016). Resveratrol reverses functional Chagas heart disease in mice. *PLoS Pathogens*.

[73] Villena J. A. (2015). New insights into PGC-1 coactivators: redefining their role in the regulation of mitochondrial function and beyond. *The FEBS Journal*.

[74] Repossi G., Das U. N., Eynard A. R. (2020). Molecular basis of the beneficial actions of resveratrol. *Archives of Medical Research*.

[75] Mukherjee S., Belbin T. J., Spray D. C. (2003). Microarray analysis of changes in gene expression in a murine model of chronic chagasic cardiomyopathy. *Parasitology Research*.

[76] Wen J., Dhiman M., Whorton E., Garg N. (2008). Tissue-specific oxidative imbalance and mitochondrial dysfunction during Trypanosoma cruzi infection in mice. *Microbes and Infection*.

[77] Wen J. J., Garg N. J. (2008). Mitochondrial generation of reactive oxygen species is enhanced at the Q_o_ site of the complex III in the myocardium of Trypanosoma cruzi-infected mice: beneficial effects of an antioxidant. *Journal of Bioenergetics and Biomembranes*.

[78] Wen J. (2017). Inhibition of NFE2L2-antioxidant response element pathway by mitochondrial reactive oxygen species contributes to development of cardiomyopathy and left ventricular dysfunction in Chagas disease. *Antioxidants & Redox Signaling*.

[79] Wen J. J., Garg N. J. (2018). Manganese superoxide dismutase deficiency exacerbates the mitochondrial ROS production and oxidative damage in Chagas disease. *PLoS Neglected Tropical Diseases*.

[80] Wan X., Wen J.-j., Koo S.-J., Liang L. Y., Garg N. J. (2016). SIRT1-PGC1*α*-NF*κ*B pathway of oxidative and inflammatory stress during Trypanosoma cruzi infection: benefits of SIRT1-targeted therapy in improving heart function in Chagas disease. *PLoS Pathogens*.

[81] Ren Z., He H., Zuo Z., Xu Z., Wei Z., Deng J. (2019). The role of different SIRT1-mediated signaling pathways in toxic injury. *Cellular & Molecular Biology Letters*.

[82] Rose E., Carvalho J. L., Hecht M. (2020). Mechanisms of DNA repair in Trypanosoma cruzi: What do we know so far?. *DNA Repair*.

[83] Maçao L. B., Filho D. W., Pedrosa R. C. (2007). Antioxidant therapy attenuates oxidative stress in chronic cardiopathy associated with Chagas’ disease. *International Journal of Cardiology*.

[84] de Souza A. P., Jelicks L. A., Tanowitz H. B. (2010). The benefits of using selenium in the treatment of Chagas disease: prevention of right ventricle chamber dilatation and reversion of Trypanosoma cruzi-induced acute and chronic cardiomyopathy in mice. *Memorias do Instituto Oswaldo Cruz*.

[85] de Souza A. P., Sieberg R., Li H. (2010). The role of selenium in intestinal motility and morphology in a murine model of Typanosoma cruzi infection. *Parasitology Research*.

[86] Ribeiro V., Dias N., Paiva T. (2020). Current trends in the pharmacological management of Chagas disease. *International Journal for Parasitology: Drugs and Drug Resistance*.

[87] Repolês B. M., Machado C. R., Florentino P. T. V. (2020). DNA lesions and repair in trypanosomatids infection. *Genetics and Molecular Biology*.

[88] Çağlayan M. (2020). Pol *β* gap filling, DNA ligation and substrate-product channeling during base excision repair opposite oxidized 5-methylcytosine modifications. *DNA Repair*.

[89] Passos-Silva D. G., Rajão M. A., Nascimento de Aguiar P. H., Vieira-da-Rocha J. P., Machado C. R., Furtado C. (2010). Overview of DNA repair in Trypanosoma cruzi, Trypanosoma brucei, and Leishmania major. *Journal of Nucleic Acids*.

[90] El-Sayed N. (2005). The genome sequence of Trypanosoma cruzi, etiologic agent of Chagas disease. *Science*.

[91] Grisard E. C., Teixeira S. M. R., de Almeida L. G. P. (2014). Trypanosoma cruzi clone Dm28c draft genome sequence. *Genome Announcements*.

[92] Furtado C., Kunrath-Lima M., Rajão M. A. (2012). Functional characterization of 8-oxoguanine DNA glycosylase of Trypanosoma cruzi. *PLoS One*.

[93] Cabrera G., Barría C., Fernández C. (2011). DNA repair BER pathway inhibition increases cell death caused by oxidative DNA damage in Trypanosoma cruzi. *Journal of Cellular Biochemistry*.

[94] Schamber-Reis B. L. F., Nardelli S., Régis-Silva C. G. (2012). DNA polymerase beta from Trypanosoma cruzi is involved in kinetoplast DNA replication and repair of oxidative lesions. *Molecular and Biochemical Parasitology*.

[95] Maldonado E., Rojas D. A., Moreira-Ramos S. (2015). Expression, purification, and biochemical characterization of recombinant DNA polymerase beta of the Trypanosoma cruzi TcI lineage: requirement of additional factors and detection of phosphorylation of the native form. *Parasitology Research*.

[96] Rojas D. A., Urbina F., Moreira-Ramos S. (2018). Endogenous overexpression of an active phosphorylated form of DNA polymerase *β* under oxidative stress in Trypanosoma cruzi. *PLOS Neglected Tropical Diseases*.

[97] Rajão M. A., Passos-Silva D. G., DaRocha W. D. (2009). DNA polymerase kappa from Trypanosoma cruzi localizes to the mitochondria, bypasses 8-oxoguanine lesions and performs DNA synthesis in a recombination intermediate. *Molecular Microbiology*.

[98] Rajão M. A., Furtado C., Alves C. L. (2014). Unveiling benznidazole’s mechanism of action through overexpression of DNA repair proteins in Trypanosoma cruzi. *Environmental and Molecular Mutagenesis*.

[99] Augusto-Pinto L., Bartholomeu D. C., Teixeira S. M. R., Pena S. D. J., Machado C. R. (2001). Molecular cloning and characterization of the DNA mismatch repair gene class 2 from the Trypanosoma cruzi. *Gene*.

[100] Campos P. (2011). Trypanosoma cruzi MSH2: functional analyses on different parasite strains provide evidences for a role on the oxidative stress response. *Molecular and Biochemical Parasitology*.

[101] Augusto-Pinto L., Teixeira S. M., Pena S. D., Machado C. R. (2003). Single-nucleotide polymorphisms of the Trypanosoma cruzi MSH2 gene support the existence of three phylogenetic lineages presenting differences in mismatch-repair efficiency. *Genetics*.

[102] Valenzuela L., Sepúlveda S., Ponce I., Galanti N., Cabrera G. (2018). The overexpression of TcAP1 endonuclease confers resistance to infective Trypanosoma cruzi trypomastigotes against oxidative DNA damage. *Journal of Cellular Biochemistry*.

